# Association study of *Bif-1* gene expression with histopathological characteristics and hormone receptors in breast cancer

**DOI:** 10.1186/s12905-022-02075-4

**Published:** 2022-11-24

**Authors:** Kazhaleh Mohammadi, Mahdieh Salimi, S. Abdolhamid Angaji, Arthur Saniotis, Foroozandeh Mahjoobi

**Affiliations:** 1 Department of Pharmacy, College of Pharmacy, Knowledge University, Erbil, 44001 Iraq; 2 Department of Medical Genetic, Institute of Medical Biotechnology, National Institute of Genetic Engineering and Biotechnology, Tehran, Iran; 3 Department of Cell and Molecular Biology Sciences, Kharazmi University, Tehran, Iran; 4 Bachelors of Doctor Assistant Department, DDT College of Medicine, Gaborone, Botswana; 5 Biological and Comparative Anatomy Research Unit, School of Biomedicine, The University of Adelaide, Adelaide, Australia

**Keywords:** Breast cancer, *Bif-1*, Gene expression, Real-time PCR, Biomarker

## Abstract

**Background:**

Breast cancer is a heterogeneous disease that has various clinical outcomes. Bax-interacting factor-1 (*Bif*-1) is a member of the endophilin B family that generates the pro-apoptotic BCL2-Associated X (BAX) protein in response to apoptotic signals. Lack of *Bif*-1 inhibits the intrinsic pathway of apoptosis and enhancements the risk of tumor genesis. The present study aimed to investigate the relationship between hormone receptors (ER, PR, and HER2) status and different levels of *Bif-1* gene expression in breast cancer patients.

**Methods:**

*Bif-1* gene expression was evaluated in 50 breast cancer tumors and 50 normal breast mammary tissues using the SYBR Green real-time RT-PCR technique. Multivariate and univariate analyses were used to appraise the relationship between the prognostic significance of the Bif-1 gene using SPSS software. In this study, the *Bif-1* was selected as a candidate for a molecular biomarker and its expression status in breast cancer patients with hormone receptors (ER, RR, and HER2) compared to patients without these hormone receptors.

**Results:**

The study showed that the relative expression of the *Bif-1* gene in tissues of patients with hormone receptors in breast cancer compared to those without hormone receptors was not statistically significant. The expression levels of the *Bif-1* gene in different groups were evaluated for hormone receptor status. No significant relationship was found between the *Bif-1* gene expression and hormone receptors (ER, PR, and HER2) (*p* > 0.05).

**Conclusion:**

*Bif-1* gene expression may be a useful prognostic marker in breast cancer.

## Background

Breast cancer (BC) is a complex heterogeneous disease due to a combination of genetic and epigenetic factors that eventually alter molecular and cellular processes consisting of proliferation, apoptosis, and angiogenesis [1, 2]. Breast cancer is the major cause of cancer death in women worldwide, with an estimated 2 million new cases diagnosed in 2018 [3]. As a consequence, BC is a major universal challenge [4].

Several studies have announced that BC is often triggered by an over-expression of biomarkers that are most commonly defined by estrogen-receptor (ER), progesterone receptor (PR), and Human Epidermal growth factor Receptor 2 (HER2) status. Estrogen-receptor and PR markers have been demonstrated to be important prognostic factors for endocrine therapy [5, 6], whereas, the HER2 gene facilitates the onset, growth, and metastasis of breast cancer [7]. There are several factors involved in cancer, containing tumor suppressor oncogenes and genes. Any variation from their normal activity may lead to unnecessary cell division and apoptotic evasion. Amongst these tumor suppressor genes is the BCL2-Associated X (BAX) interacting factor-1 (*Bif*-1) gene. The *Bif-1* gene can induce autophagy, a process that can protect normal cells by conserving intracellular homeostasis [8]. This process of autophagy involves the interaction of *Bif-1* with the protein Beclin-1 via the facilitation of Ultraviolet irradiation resistant-associated gene (UVRAG) that controls phosphatidylinositol 3-kinase complex 3 (PI3KC3) [9]. Contemporary therapies rely on the expression of ER and HER2 receptors for the treatment of Triple Negative Breast Cancer. Unfortunately, these have proven to be ineffectual for the treatment of metastatic carcinoma. Hence, there is a need for the development of new therapies based on molecular biomarkers that will be discussed. In the present study, *Bif*-1 was selected as a candidate for a molecular biomarker and its expression status in breast cancer patients with hormone receptors (ER, PR, HER2) compared to patients without these hormone receptors. Patients with breast carcinoma mostly have elevated sensitivity to hormone-based therapy if they have high PR and ER [10]. Researchers have long sought to develop primary diagnostic methods for recognizing cancer. Advances in the use of biomarkers have been effective in the diagnosis and treatment of breast cancer and have led to the application of some of these markers in patients. Popular methods for measuring biomarkers such as immunohistochemistry, immunocytochemistry, and ELISA are widely accepted and repeated methods in various laboratories [11]. However, the non-quantitative results and the innumerable and lengthy workflows are limitations of these techniques. Consequently, this has prompted researchers to search for alternative molecular methods such as Quantitative Real-Time PCR (Q-RT-PCR) that detect DNA amplification [12–16]. The Q-RT-PCR technique is a rapid, cost-effective, and high-specificity assay for the assessment of gene expression [12]. Q-RT-PCR technique has significant potential in biomarker detection relating to mammaglobin (MGB) and metastasis of the lymph nodes [17]. For example, it has been recently shown that RT-PCR had a considerable diagnostic prediction for detecting mammaglobin biomarkers for lymph node metastasis in breast cancer patients [17].

Final verification of this method for use in diagnostic laboratories requires numerous validations including housekeeping genes. Several large-scale gene expression studies have been accomplished in which hundreds of housekeeping genes have been identified [18–20].

Dysregulation of *Bif-1* expression in cancer cells compared to adjacent healthy tissue, in many types of cancer including colorectal cancer [21], prostate [22], pancreatic cancer [23], invasive bladder cancer [24], and stomach cancer [25] have been observed. Decreased expression of *Bif-1* in epithelial cells in malignant gastric cancer compared to normal mucosal cells suggests that loss of *Bif-1* expression may play a role in gastric tumorigenesis by inhibiting *Bif-1* mediated apoptosis [25]. On the other hand, Takahashi et al. showed in a study in 2007 that suppressing *Bif-1* in mice promotes tumor progression [26]. In a study on colorectal cancer, it was seen that patients with low levels of *Bif-1* in stages I and II of the disease have a lower chance of survival. This suggests that *Bif-1* protein expression may be a useful prognostic marker in the early stages of colorectal cancer. Loss of the *Bif-1* tumor suppressor gene is involved in various types of tumors and cancers [27].

In this study, the *Bif-1* gene was selected as a candidate for a molecular biomarker and its expression in normal individuals and breast cancer patients was evaluated. The *Bif-1* gene was examined according to its histopathological characteristics (stage and grade status) in patients’ tumors, as well as the expression status of hormonal receptors (ER, RR, HER2) which have prognostic value.

This study aimed to confirm the role of the *Bif-1* gene as a predictor of breast cancer for better and proper management of training. Moreover, our study of molecular biomarkers, including the *Bif-1* gene, could pave the way for biomarkers with therapeutic value shortly; second, it could lead to the production of effective drugs for the treatment of breast cancer patients and especially those patients with Triple Negative Breast Cancer.

## Methods

### Patient information

Totally, 100 samples of breast cancer patients, fifty cases relevant to breast tumor tissues (during primary diagnosis and without chemotherapy or radiation therapy at the time of sampling) along with 50 adjacent normal tumor specimens, from Milad and Khatam al-Anbia Hospitals, Tehran, Iran, were selected by following ethical rules in Helsinki Experimental Medical Studies (Declaration of Helsinki (DoH)). All the patients signed informed consent. The ethics code number is IR.NIGEB.EC.1395.11.10. I. The average age of studied patients was 50.0 ± 10.5 in the 30–70 age range (years). The inclusion criteria were the TNM classification system and post-operative diagnosis of primary BC based on histopathology. The characteristics of our studied patients are shown in Table 1. Overall survival (OS) was defined as the length of time from the date of primary diagnosis for patients with BC to the date of death from any cause. Disease-free survival (DFS) for patients with BC was defined as the time from the date of primary diagnosis to the recurrence of the tumor or the date of death. Samples were transferred (Transferred to the tank containing liquid nitrogen) to the National Institute of Genetic Engineering and Biotechnology, Tehran, Iran, under the principles of transfer and maintenance. Then, all achieved samples were preserved in a freezer at − 70 °C for < 2 h.

**Table 1:** Baseline characteristics of breast cancer patients

Characteristic	Number (%)
Number of patients	100
Age (years, mean ± SD)	50 ± 10.57691
Range	30–70
Cancer type	
Ductal carcinoma	32 (64%)
Lobular carcinoma	10 (20%)
Ductal and lobular carcinoma	5 (10%)
Unknown	3 (6%)
Lymph node status	
N0	3 (6%)
N+	27 (54%)
Nx	20 (40%)
Pathological stage	
Stage I	9 (18%)
Stage II	36 (72%)
Stage III	5 (10%)
Hormone-receptor status (IHC)	
ER and/or PR positive	35 (70%)
ER and/or PR negative	9 (18%)
Unknown	6 (12%)
HER-2 status (IHC)	
HER-2 positive	23 (46%)
HER-2 negative	21 (42%)
Unknown^a^	6 (12%)
Triple Negative Breast Cancer (HER-2^−^, PR^−^, ER^−^)	5 (10%)

^a^In 6 of the studied patients, breast cancer stage was determined based on the old TNM classification system (before 2018). In these patients, based on the primary tumor size (T), the regional lymph nodes status (N) were taken into consideration by the attending physician. For this reason, the status of hormone receptors in these patients was reported to be unclear

### RNA extraction and cDNA synthesis

Total RNA was isolated from the breast tissue using TriPure Isolation Reagent (Roche) solution according to the manufacturer’s protocol. cDNA was synthesized following the manufacturer’s instructions (Revert AID First strand cDNA synthesis Kit) and stored at − 20 °C until analyzed. The housekeeping gene *β-actin*, generally used in gene expression studies in breast cancer, was selected as an internal control. The expression stability of the *β-actin* gene data was evaluated to normalize the *Bif-1* gene expression data. The primer sequences for *β-actin* and *Bif-1* genes were designed using primer 3 software (https://primer3.ut.ee/) and then blasted using https://www.ncbi.nlm.nih.gov/tools/primer-blast/. The information on designed primer sequences is presented in Table 2. The duplication efficiency of each primer was determined using the standard curve of one-tenth of the cDNA serial dilutions using SPSS (LinReg, Statistical Package for Social Science IL, USA, V.16, SPSS Inc., Chicago) software. The cDNA used for serial dilution of a mixture of 15 tumor samples was equal in proportion.

**Table 2:** Characteristics of primers used in real time RT-PCR reaction

Gene name	Primer sequence	Product size (bp)	Annealing temperature
*Bif-1*	F:5′CTAGAGGGAATCAGCAGTACACATG3′	174	60.74
	R:5′AGGTGTCACAGAAGTCTGATTGTTG3′		61.20
*β-actin*	F:5′ GAGACCTTCAACACCCCAGCC 3′	161	62.93
	R:5′ AGACGCAGGATGGCATGGG 3′		62.41

Tumor tissue samples and the tissue adjacent to the tumor lesion from patients were tested using Real-Time PCR. In this step, for normalization, the *β-actin* gene was used. In all, the expression of the *β-actin* gene in the normal tissue around the tumor was investigated. This was conducted to obtain control for distinguishing normal expression from overexpression of this gene.

The Real-time RT-PCR was done following the manufacturer’s instructions (Roche Applied SYBR Green I Master Mix Kit Science 480). The Real-time RT-PCR amplifications were conducted in a final volume of 10 μl reaction mixture containing 1 μl of cDNA, 2 μl 5 × master mix green (FastStart Taq DNA Polymerase, reaction buffer. dNTP MIX (with dUTP instead of dTTP), SYBR dTTP, SYBR Green 1 dye and Mgcl2), 0.3 μl (10 pmol/μl) of each primer and 6.4 μl sterilized water, using the Rotor-Gene Q System (QIAGEN Hilden, Germany). Table 3 shows the Real-time RT-PCR cycling conditions.

**Table 3:** Real-time RT-PCR cycling conditions for *Bif-1* and *β-actin* gene

Step	Cycle number	Temperature (C°)	Duration
Primitive denatured	1	95	10'
Denatured		95	20''
Connecting primers	40	62	15''
Expansion of primers		72	15''
Melting stage or temperature gradient of 72 to 95 (C°)	1	95	5"

For each data point, all experiments were repeated three times. For the amplification efficiency determination of each primer pair, the ultraviolet spectrophotometer was used for assessing the linear standard curve (from 0.1 to 1000 ng). The acceptable linearity and amplification (100%) were revealed via the standard curves.

### Bif-1 Expression in tumor and adjacent normal tissues in breast cancer patients

Tumor immune estimation resource (TIMER) (http://cistrome.shinyapps.io/timer) [28] was adopted to infer the differential expression between the tumor and adjacent normal tissues for the *Bif-1* gene in breast cancer. As for protein level, the cProSite database (Cancer Proteogenomic Data Analysis Site), was used to compare the protein abundance of *Bif-1* between tumor and normal adjacent tissues.

### Bif-1(SHEGLB1) Expression and ICI

Tumor immune estimation resource (TIMER) also was used to infer the immune cell infiltration (ICI) and its relations with *Bif-1* in breast cancer patients.

### Statistical analysis

The raw data from Real Time RT-PCR were analyzed by LinRrg software and reproduction efficiency and CT numbers were obtained for each reaction. Next, the expression changes of the studied genes were evaluated by LinReg software using LinReg output. To obtain the difference in expression of the target genes and the reference gene, the Livak 2^−ΔΔCT^ method was used [29]. Finally, the data analysis and statistical analysis were accomplished via SPSS software version 16 was used and as well as using the Kolmogorov Smirnov test, the normality of the achieved data was checked. Since the data distribution was not normal nonparametric tests such as Kruskal Wallis and the Mann–Whitney U tests were used for numerical data and to compare the expression of genes with response to treatment and clinical symptoms Chi-square test was performed. The confidence interval was 95% in all experiments and *P* < 0.05 was considered significant.

## Results

### Bif-1 expression and clinic pathological features

#### Bif-1 mRNA Expression

The aforementioned institute adhered to all ethical guidelines relating to biological banks for the preservation and use of human specimens. ER, PR, and HER2/neu biomarkers were evaluated in 44 patients, with positive or negative biomarkers, which are listed in (Table 1).

All the data as mentioned were normalized using *β-actin* as an internal control and normalizer and then presented relative to the average expression level in normal control breast tissue using the formula 2^−ΔΔCT^. RNA expression with two times or more was considered as overexpression, between 0.5 and 2 times as normal, and 0.5 times and less as down expression. Finally, 68% of the data showed the down expression (˂ 0.5), 20% the normal expression (0.5˂–˂2), and 12% the overexpression (2≤–˂10). In breast cancerous tissues compared with normal control, the *Bif-1* mRNA expression was significantly downregulated (*P* < 0.01). In cancerous tissues compared with normal control, the mean of *Bif-1* relative expression was 0.71 ± 1.03 with a range of 0.0001 to 3.98. About 68% of cancerous samples showed a relative expression of < 0.5 which was considered downregulation. The low average expression in tumor tissue compared to the adjacent normal tumor confirms the tumor suppressor function of the *Bif-1* gene.

#### Bif-1 expression in tumor and adjacent normal tissues

**Fig. 1:**
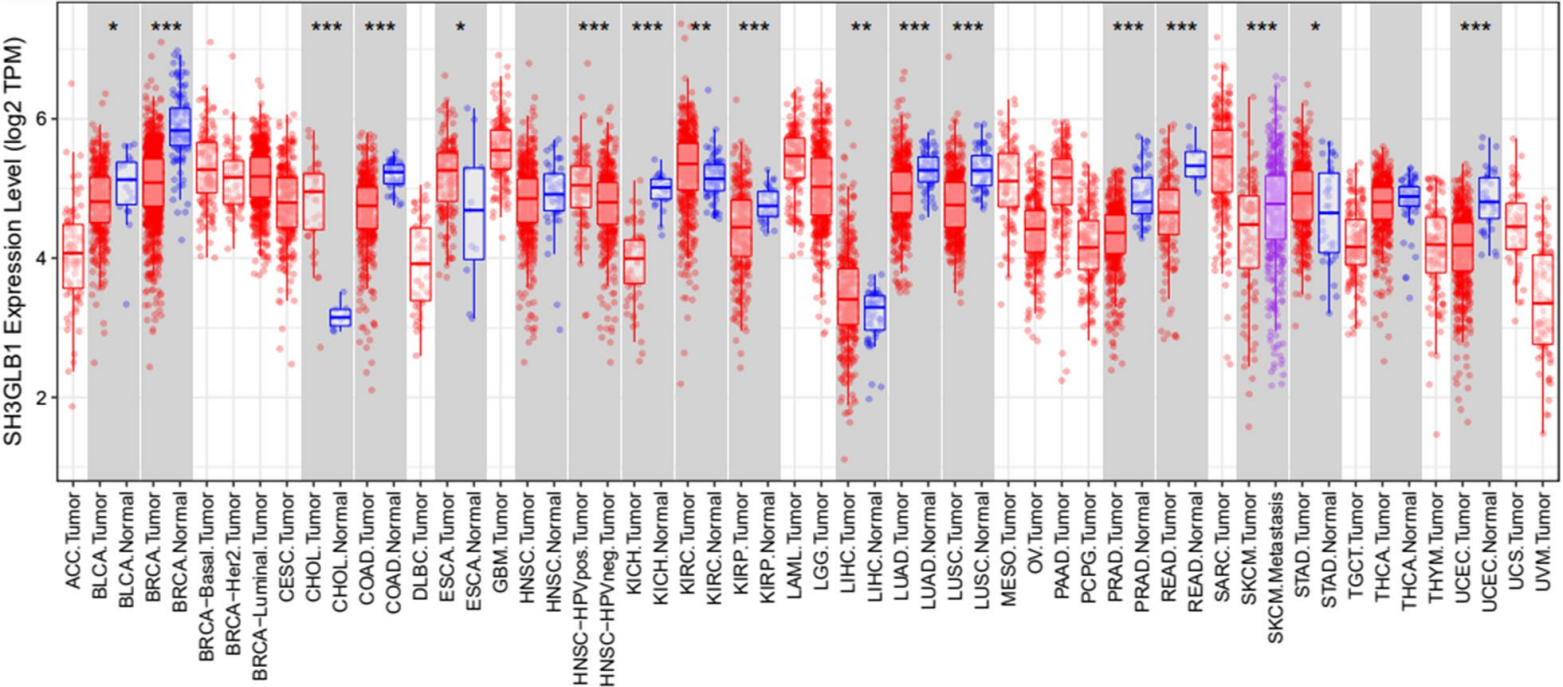
The differential expression between tumor and adjacent normal tissues for the *Bif-1* gene across all TCGA tumors based on the DiffExp module in TIMER (Tumor IMmune Estimation Resource).In Tumor IMmune Estimation Resource, normal data for some types of cancer has been displayed in gray columns are available. In breast cancer patients, the *Bif-1* expression level is highest in normal tissue and least in cancerous tissues

On other hand, the differential expression between the tumor and adjacent normal tissues for the *Bif-1* gene across all TCGA tumors using the DiffExp module in TIMER (Tumor IMmune Estimation Resource) database was achieved. *Bif-1* gene expression levels has been distributed using box plots, with the statistical significance of differential expression evaluated using the Wilcoxon test. The current findings also showed that *Bif-1* gene mRNA in breast normal tissues compared to breast cancerous tissues comprised a higher level of expression (Fig. 1).

### Bif-1 protein expression

**Fig. 2:**
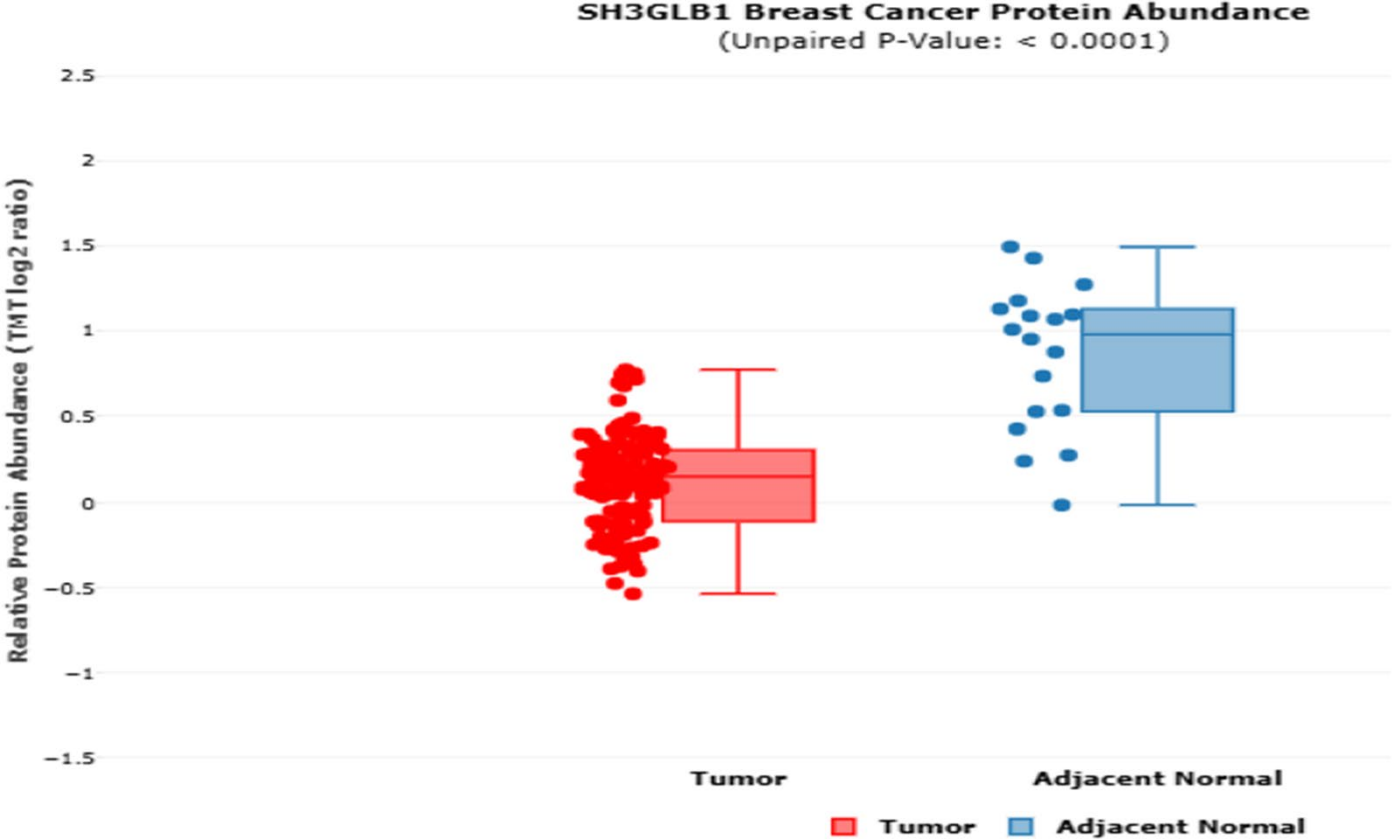
*Bif-1* protein expression in tumor and adjacent normal tissues in breast cancer patients based on cProSite database. *Bif-1* protein abundance in BC tissues is lower than the normal adjacent tissues

As for protein level, the cProSite database (Cancer Proteogenomic Data Analysis Site), was used to compare the protein abundance of *Bif-1* between tumor and normal adjacent tissues. The *Bif-1* protein abundance in BC tissues compared to normal adjacent tissues was lower level (*p*  < 0.05, Fig. 2).

**Fig. 3:**
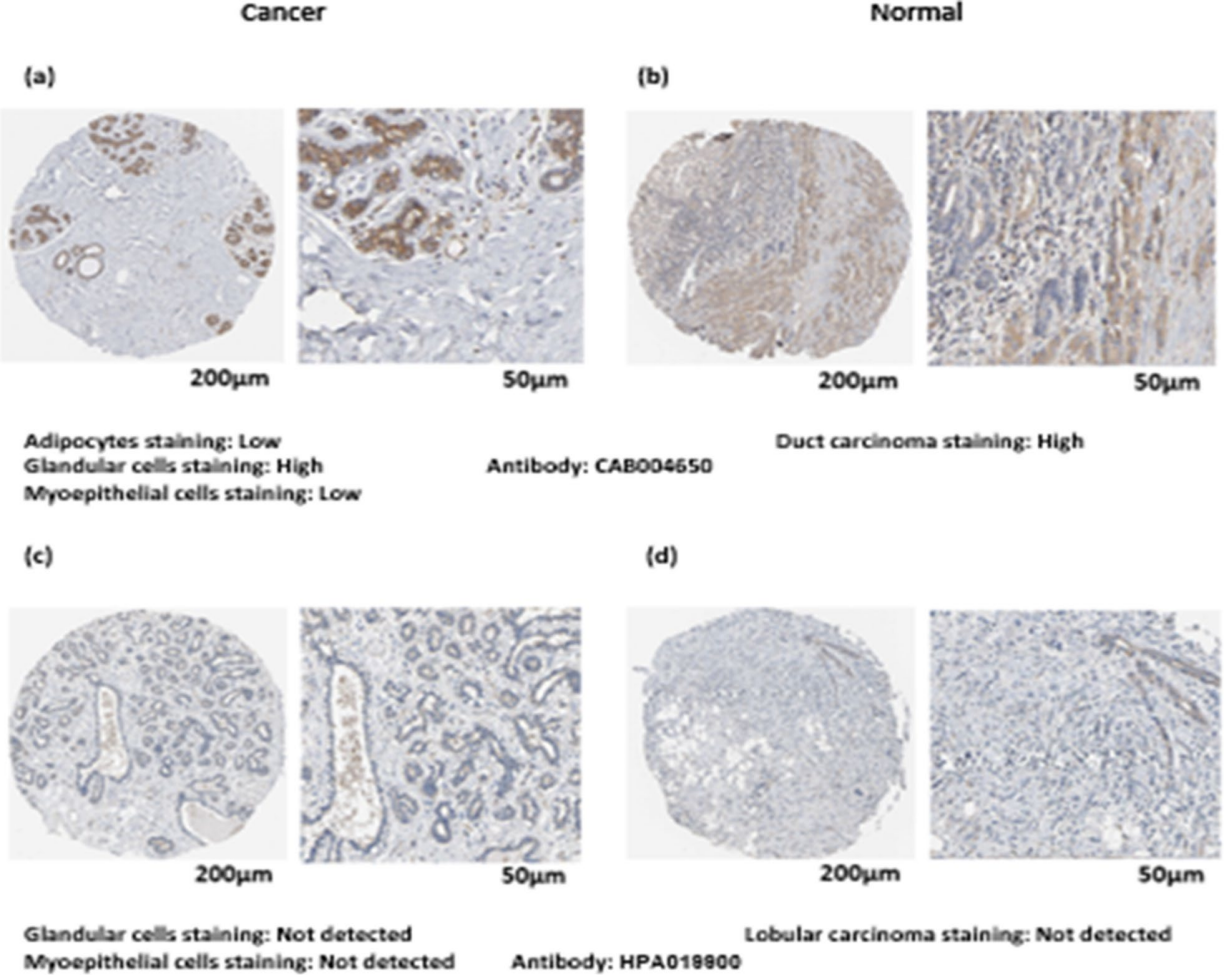
IHC analysis of *Bif-1(SH3GLB1)* in Breast cancer. **a**–**d** *Bif-1* protein expression level in normal tissue and duct & lobular carcinoma tissue has been detected based on human protein atlas database

Based on the Human Protein Atlas database, immunohistochemical staining of clinical specimens has identified that the *Bif-1* expression level in BC tissues compared to adjacent normal tissues is lower (Fig. 3a–d).

**Fig. 4:**
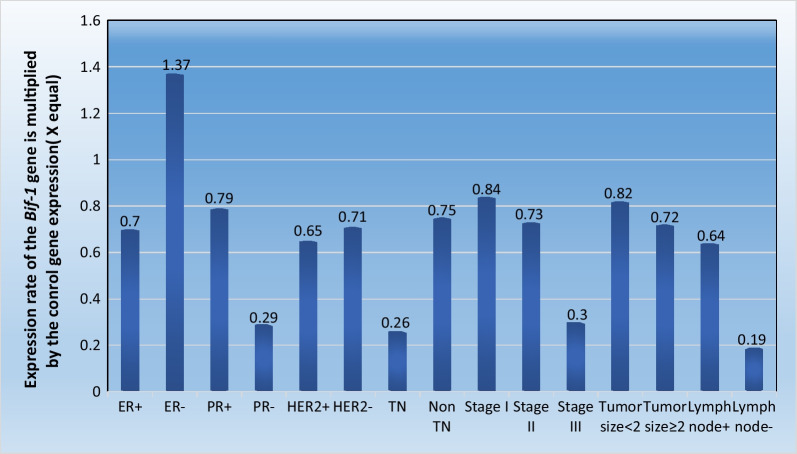
Average expression of the *Bif-1* gene in different tumor groups. All data were normalized using *β-actin* as an internal control and normalizer and presented relative to the average expression level in normal control breast tissue using formula was 2^−ΔΔCT^. (ER; estrogen receptor, PR; progesterone receptor, HER_2;_ Human epidermal growth factor 2, TN; Triple Negative, non-TN non; Triple Negative)

In the present study also, the expression levels of the *Bif-1* gene in different groups were evaluated as hormone receptor status, disease stage, lymph node involvement, different types of breast cancer and, tumor size states. No significant differences showed between other demographical and clinic pathological characteristics of breast cancer patients and Bif-1 expression (*P* > 0.05), as shown in Fig. 4.

The current findings showed that there was no statistically significant relationship between *Bif-1* gene expression and disease stage (*p* > 0.05) and lymph node involvement in breast cancer patients (*p* > 0.05). Alternatively, there was no statistically significant relationship between Bif-1 gene expression and different types of breast cancer (ductal, lobular, and ductal & lobular carcinoma) (*p* > 0.05). Regarding tumor size, 32 patients (64%) had a tumor equal to or more than two centimeters, and 18 patients (36%) had a tumor size less than two centimeters. According to the results of linear regression, there was no significant relationship between gene expression and tumor size in breast cancer patients (*P* > 0.05). Concerning hormone receptor status, our findings indicate that the expression of *Bif-1* was increased in patients who have at least one hormone receptor. However, expression of the *Bif-1* gene was reduced in patients with triple-negative hormone receptors. There was no statistically significant relationship between *Bif-1* gene expression and all three hormone receptors (ER, PR, HER2) (*P* > 0.05).

#### Link between Bif-1 and ICIs

**Fig. 5:**
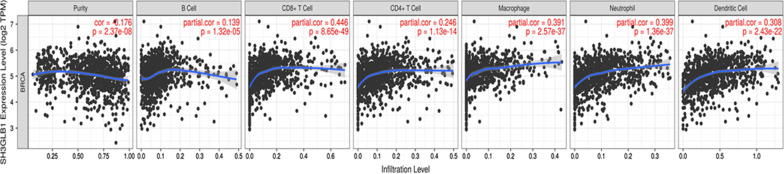
The link between *Bif-1* expression and ICIs (B cells, CD8 + T cells, CD4 + T cells, macrophages, neutrophils, and DCs) in breast cancer patients based on TIMER databas

The correlation of Bif-1 gene expression with immune infiltration levels in breast cancer was achieved based on the Gene module in TIMER database (Fig. 5). The link between *Bif*-1 expression and ICIs was adjusted by purity, B cells, CD8+ T cells, CD4+ T cells, macrophages, neutrophils, and DCs and was studied by TIMER. In breast cancer, the results proved that *Bif-1* expression was positively related to the infiltration of purity (r = − 0.176, 2.37e−08), B cells (r = 0.139, *p* = 1.32e−05), CD8+ T (r = 0.446, *p* = 8.65e−49), CD4+ T cells (r = 0.246, *p* = 1.13e−14), macrophages (r = 0.391, *p* = 2.57e−37), neutrophils (r = 0.399, *p* = 1.36e37), and DCs (r = 0.308, *p* = 2.43e−22).

## Discussion and conclusion

Cancer is the main cause of death in economically developed countries and the second cause of death in developing countries [30]. The World Health Organization estimates that cancer kills about seven million people worldwide every year [31]. Therefore, finding suitable treatment methods is one of the strategies of researchers to increase the survival of these patients, which is done by finding suitable markers to evaluate the results of treatment (prognostic factors) and also markers of response to treatment. In this study, *Bif-1* gene expression (one of the genes involved in causing cancer) in breast cancer and its relationship with histopathological characteristics (stage) and tumor grade and hormone receptors (PR, ER, HER2) in patients with breast cancer, was investigated.

In the present study, the expression of *Bif-1* at mRNA and protein levels was significantly decreased in BC tumors compared with normal control. Meanwhile, there was no association between the *Bif-1* gene expression and lymphatic metastasis. In other hand, there was no statistically significant relationship between *Bif-1* gene expression and estrogen(ER), progesterone(PR), and human epidermal growth factor receptor 2(HER2) hormone receptors.

Unfortunately, few studies have been conducted to quantitatively evaluate the expression of *Bif-1*, and some studies of *Bif-1* gene activity, have been inconsistent. In addition, several studies have compared the expression of *Bif-1* in patients' tumor cell lines with that of the control group [32]. We contend that this is not a tenable measure for evaluating both tumor tissue of patients and normal tissue samples. It should be noted that, for investigation, it is better to test the patient's tumor tissue sample and their adjacent normal tumor sample, and our results support this case.

To our knowledge, this study is the first to investigate the relationship between hormone receptor status (ER, PR HER2) and different levels of *Bif-1* gene expression in patients with breast cancer. However, *Bif-1* can also act as a tumor suppressor because of its role in regulating the BAX gene. *Bif-1* accelerates BAX degradation directly by binding to BAX and enhancing apoptosis induction kinetics in response to innate apoptotic signals, thereby increasing the permeability of the mitochondrial outer membrane. Previous studies correspond with our findings, for example, Cuddeback et al. reported that the *Bif-1* protein was silent in 17% (192.33 patients) of all prostate cancer patients. These findings indicate the activity of tumor suppression and pro-apoptotic *Bif-1* [33]. Impaired expression of *Bif-1* in cancer cells, compared to adjacent normal tissue, has been observed in various types of cancer, including colorectal cancer [21], prostate cancer [22], pancreatic cancer [23], invasive bladder cancer [24], and gastric cancer [25].

In another study, Coppola et al. found that *Bif-1* lacked expression in approximately 45% of patients with malignant pancreatic cancer, but had a high level of expression in patients with benign pancreatic cancer [23]. Similarly, Fan et al., observed that patients with high *Bif-1* expression compared to patients with low *Bif-1* gene expression, had a shorter survival time, their study correlates *Bif-1* expression to survival time [34].

Takahashi et al. showed that suppression of the *Bif-1* gene in mice promoted tumor progression which corresponds with our study’s findings. In this study, it was observed that the *Bif-1* gene expression decreased in patients with tumor size equal to or more than two centimeters, and vice versa, expression of this gene increased in patients with tumor size less than two centimeters. This result shows the induction function of the *Bif-1* gene apoptosis [26].

As indicated before, having a reference method for examining the expression of this gene to study its role in the development of various cancers is necessary. Among the major methods of studying gene expression, the Real-Time RT-PCR technique provides the highest sensitivity and accuracy for determining the expression level. To study the expression of genes in clinical samples of patients, it is necessary to use a sensitive method (E.g. Real-Time RT-PCR technique), because the expression of genes in clinical samples is less than that of cell lines.

## Conclusion

Until now, the information obtained from molecular markers has been inadequate, so identifying the convenient biomarkers in the evaluation of treatment response in patients with breast cancer is important. In all cancer cells, *the Bif-1* gene has a low expression or is turned off (in 80% of cancers this gene has a low expression or is turned off) and *Bif-1* has high expression in most normal cells. For this reason, *the Bif-1* gene can be considered a promising target for the treatment of various types of cancer. Our findings note that the *Bif-1* gene expression was downregulated in breast cancer patients and, its expression in both mRNA and protein levels. Therefore, the *Bif-1* gene expression changes can be introduced as a strategic marker in breast cancer patients. Generally, increasing in the expression level of *Bif*-1 at the protein level is happen in line with the increase in mRNA level. In conclusion, our findings divulged that the *Bif-1* gene expression change can be used as a clinically relevant prognostic indicator in breast cancer. Therefore, it is suggested that *Bif-1* gene expression analyses may be important when making informed and individualized clinical decisions regarding the management of breast cancer patients. Considering the importance of this gene in breast cancer, it is expected that better and more favourable results can be obtained by using more samples. Therefore, more extensive research in this field with more samples as well as the examination of the expression of this gene in other tissues is suggested. In another way, to study the expression of genes in patients' clinical samples, it is necessary to use a sensitive method, since the expression of genes in clinical samples is less than in cell lines. Real-Time RT-PCR enables the highest sensitivity and accuracy for specifying the amount of expression. Therefore, due to the high sensitivity of the Real-Time RT-PCR method and in addition that the RNA and cDNA obtained from the cells can be stored and reused, it also seems to be faster and cheaper than the flow cytometry method. Consequently, an optimal way of evaluating *Bif-1* gene activity at the mRNA level is to use Real-Time RT-PCR.

## Data Availability

All data used and/or analyzed during the current study are available from the corresponding author upon reasonable request.
